# Use of Technological Devices in Children Aged 3–11 Years: Possible Effects on Sleep and Behavioral Difficulties

**DOI:** 10.3390/pediatric17050099

**Published:** 2025-09-29

**Authors:** Marta Tremolada, Roberta Maria Incardona, Sabrina Bonichini, Livia Taverna

**Affiliations:** 1 Department of Developmental and Social Psychology, University of Padua, 35131 Padua, Italy; robertamaria.incardona@phd.unipd.it (R.M.I.); s.bonichini@unipd.it (S.B.); 2 Faculty of Education, Free University of Bolzano-Bozen, 39100 Bolzano, Italy; livia.taverna@unibz.it

**Keywords:** children, technological devices, behavioral problems, emotional difficulties, sleep

## Abstract

Background: The use of technological devices by children has increased in recent years, as several Italian studies have shown, even if limited studies assessed the possible effects on sleep and psychological wellbeing in preschool and schoolchildren. Objectives: This cross-sectional study seeks to examine the prevalence and typology of usage, as well as the potential socio-demographic factors associated with it. Additionally, it aims to assess emotional behavior and sleeping difficulties, and their possible correlations with children’s attitudes towards technological devices. Methods: The study included 314 children (52.2% males), with an average age of 7.86 years (SD = 2.02) and mainly their mothers (80.2%). Participants were given a series of self- and proxy-report questionnaires about the digital age, type of activities, hours of sleep, and their behavioral/emotional difficulties (CBCL and SDQ questionnaires). The analyses were descriptive and correlational because of the explorative nature of this study. Results: Parents declared tablets as the device most used by children (49.8%), with an average daily use time of 22.86 min (SD = 33.62). Their digital starting age was on average 5.91 years (SD = 2.25) with mostly recreational activities (Mean = 3.15; SD = 0.72). Parents and children agreed on the time spent with the technological devices reports, while they discorded on the means of creative activities use (t_109_ = −8.86; *p* < 0.001), with children reporting a higher frequency (M = 2.45; SD = 0.69) than their parents (M = 1.89; SD = 0.76). The tablet time was significantly different by gender (t_309_ = −2.34; *p* = 0.02), with boys using tablets for a longer mean time than girls (27.8 versus 18.22) and having more ADHD problems (t_312_ = −3.11; *p* = 0.002; 2.68 versus 1.74). Parents declared an average of 9.55 (SD = 0.55) hours of sleep per night for their children. A significant correlation was found between PC usage time and sleep hours duration (r = −0.13; *p* = 0.019). The frequency of tablet use was correlated with both the Behavioral Problems Scale (r = 0.15; *p* = 0.010) and the ADHD Scale (r = 0.11; *p* = 0.049). Conclusions: Based on these empirical results, preventive programmes and educational activities should be established.

## 1. Introduction

### 1.1. Incidence of Use of Technological Devices in Italy, Possible Positive and Negative Consequences on Child Development and Associated Factors

According to data reported by Save the Children, in Italy approximately one in three children between the ages of 6 and 10 (32.6%) use a smartphone every day, a trend that has been continuously increasing in recent years (in 2018–2019 it was 18.4%) and with a clear prevalence in the South and the Islands, where the share increases to 44.4%, more than 20 percentage points higher than 23.9% in the North [1]. In Italy, 73% of children and adolescents between 6 and 17 years of age use the Internet every day and do so mainly through smartphones and 65.9% of children and adolescents use their cell phones every day [1]. The age at which people own or use a smartphone is increasingly lower, with a significant increase after the pandemic: Children aged 6–10 who use their cell phones every day went from 17.4% to 27.6% between the two-year period 2018–2019 and 2019–2020 [1].

The swift progress of digital technologies has greatly transformed everyday life, especially among younger generations [2], shaping their cognitive, emotional, and social growth [3]. The concept of digital natives has been widely discussed in the literature to describe individuals who have grown up with digital technology as an intrinsic part of their environment [4]. Unlike digital immigrants who had to adapt to technological advancements later in life, digital natives exhibit an intuitive familiarity with digital devices. Prensky later revised his perspective by emphasising digital competence over generational distinctions [4]. The shift toward digital wisdom, as opposed to mere digital fluency, underscores the importance of fostering a conscious and responsible approach to the use of technology among young users [5].

The growing availability and frequent use of such devices sparks crucial concerns regarding their effects on children’s adjustment, behavior, and overall emotional health. The growing body of literature on the use of digital media among children highlights both the potential benefits and the associated risks, even if the experimental Italian studies are missing or limited only to incidences of use or to the effects of the adolescents’ social network use. Concerns have emerged regarding the potential negative effects of the excessive use of digital media. Psychological and behavioral outcomes, including identity formation, emotional regulation, and maladaptive behaviors could be associated with excessive screen time [6]. Prolonged screen time has been associated with issues such as reduced sleep duration [7], increased risk of attention difficulties [8], and lower social and academic competencies [9]. Dworak et al. [10] found an association between excessive use of television and video games and emotional and behavioral symptoms. Sanders et al. [7] reported an association between less screen time and fewer externalizing, as well as internalizing, problems. Furthermore, the potential risks of exposure to the Internet, including cyberbullying and privacy concerns, emphasise the need for parental mediation. However, digital technologies have been shown to facilitate learning, creativity, and social connectivity. Research suggests that exposure to interactive digital media can improve cognitive skills, support the acquisition of a second language, and improve information-processing skills [11]. Furthermore, the incorporation of digital tools within educational environments is becoming increasingly prevalent, with the aim of enhancing student engagement and knowledge retention through interactive and dynamic learning experiences that extend beyond conventional teaching methods. In addition, the integration of technology not only enhances lesson engagement but also cultivates essential digital literacy skills, preparing students for a world where technology is integral to both the personal and professional spheres [12].

Several different factors influence the effect of technology use, such as parental participation and individual understanding of how best to use technology, and it is important to consider these parameters. Other factors such as gender could impact the time spent using technology and the different type of use. Gender differences in digital participation have also been observed, with boys generally engaging more in games and online purchases, whereas girls tend to use digital devices primarily for communication and social interaction [13]. Parental monitoring can counteract the negative effects of technology use, and can make a difference. Co-viewing and co-engaging with children while they use technology can enhance their learning experiences and promote positive interactions [14]. Technology integration should be aligned with developmentally appropriate practices, considering children’s individual needs, cultural backgrounds, and developmental stages [15]. Children of both parents with less education watched more television; children of fathers who had some college education or graduated from college spent more time on computers than those who fathers had the lowest level of education [16]. Parents had positive beliefs about technology, with smartphones being the most used among their children and the most effective device, while e-reading was the least used. The findings indicated that parents believe technology benefits their children in developing intellectually, socially, physically, and emotionally, thus enhancing children’s educational development through educational activities while interacting with peers at the same time [17]. Furthermore, it was found that there was a positive correlation between parental phubbing and children’s addiction to mobile phones [18].

### 1.2. Research Gaps and Purpose

The high incidence and the lower starting age of the use of technological devices in Italy should alert educators and psychologists on how this phenomenon could influence children’s mental health and behavioral difficulties, with particular attention given to a lower age range (3–11 years old) adopting a preventive point of view. Toddlers and first-grade schoolchildren have not received wide attention in the current studies, which were more focused on pre-adolescents and adolescents. Adopting a developmental point of view, it is essential to go in depth in the starting age of use to understand better which factors could be protective or at risk in influencing the next behaviors in the next life cycles. Considering the different use of these devices throughout the Italian country, it is important to focus on a specific cultural and regional context, such as the north-east Italian selection, characterized by high density population and industrialization degree. Different ages and regions could show different trends. This study aims to examine the current state of technological usage among schoolchildren in relation to their behavioural wellbeing in Italy, with a particular focus on the north-eastern region. Addressing this issue can yield valuable insights into how cultural norms, educational practices, and family dynamics shape children’s interactions with technology, and how these interactions affect their over all wellbeing.

The research questions were as follows.

1. What is the starting digital age of north-east Italian children and the type of use of digital devices along with self- and parental perceptions?
2. The time of technology usage and the typology of activities could differ depending on the age and gender of the children?
3. What are the possible associations between parental SES and the time and typology of technological device use?
4. What about the possible associations between the usage time of digital devices and psycho-physical health (sleep and behavioural difficulties)?

## 2. Materials and Methods

### 2.1. Research Design

This study has a cross-sectional and exploratory design to examine the prevalence and typology of the usage of technological devices, as well as the potential socio-demographic factors, emotional behaviors, and sleeping difficulties associated with it. The statistical analyses plan provides descriptive analyses on technological devices use, Pearson’s correlations to identify the possible significative associations with other linear variables (i.e., emotional behavior and sleeping difficulties scores), *t*-tests, and ANOVAS to identify the two (i.e., gender) or three levels of variables (i.e., age range) associated with the targeted examined variables.

### 2.2. Participants

The study involved 314 children (52.2% boys), with an average age of 7.86 years (SD = 2.02) and one of the parents’ couples, mainly mothers (80.2%), with an average age of 42.74 (SD = 5.36). Table 1 and Table 2 show the sociodemographic information of the children and their parents.

### 2.3. Procedures

The protocol was approved by the Psychology Ethics Committee of the University of Padua (protocol 4039). All participants provided written informed consent to participate in accordance with the Declaration of Helsinki. The consent form was then scanned and sent to the study director for further administration. The inclusion criteria were as follows: Italian language speaker, signature of the informant consent by parents, and without learning disabilities. Once signed informed consent was obtained, the link to the compilation was sent by email to the children and their parents, which was carried out online through the LimeSurvey platform. The mean time to complete the questionnaires was approximately 25 min. The participants in this study were parents and children from north-eastern Italy who completed various questionnaires from 2017 to 2020 during the Padua University courses at the Faculty of Psychology. The students of selected faculty courses contacted parents of children introducing them to the aims of the study, and they monitored the compilation of questionnaires on the LimeSurvey platform. The three different cohorts were combined for a total of 314 subjects. The questionnaires were completed by parents of children aged 3–11 years and children aged 7–11 years. Subsequently, the collected data were coded and entered into SPSS 29.0 software for statistical analysis.

### 2.4. Instruments

The adopted instruments were selected for their widespread use for screening psycho-social wellbeing in children adopting their parental observations and the possibility to compare with norms. The questionnaires on the digital experience of children and their sleep were built ad hoc for this age range adopting self- and parental perceptions.

#### 2.4.1. Sociodemographic Questionnaire

This questionnaire allowed us to collect useful information to understand the socio-economic and cultural levels of the family. The questionnaire included enquiries designed to examine the age, years of formal education, educational qualifications, and employment status of parents and partners. Additionally, it sought information on the age and gender of the child as well as the family’s demographic data, including the number of children, housing conditions, and economic status. Table 1 and Table 2 show the socio-demographic results related to this sample.

#### 2.4.2. Parent Questionnaire on Child Digital Experience (3–11 Years)

This questionnaire, completed by parents, was designed to assess the digital experiences acquired by the child. It included questions that examined the frequency of use of each digital device and the types of activities performed. Additionally, it provided information related to children’s nighttime usage. The duration of technical device usage was converted to minutes to facilitate comparisons with other variables (Table 3).

#### 2.4.3. Child’s Questionnaire on Child Digital Experience (7–11 Years)

This questionnaire is composed of the same items as the parent questionnaire on the digital experience of the child explained before. This instrument assesses the frequency of use of each digital device, the preferred ones, and the type of activity performed (Table 3).

#### 2.4.4. Child Behavior Checklist—CBCL/6-18

CBCL/6-18 is a behaviour assessment questionnaire that is part of the Achenbach System of Empirically Based Assessment (ASEBA). This tool facilitates the assessment of social skills and emotional–behavioural problems. This study focused solely on the skills scale, which is instrumental in profiling subjects through 20 items distributed across three scales: the activity scale, which measures both the extent and quality of a child’s engagement in sports, hobbies, games, and activities, as well as the quantity and quality of tasks completed and friendships developed; the sociability scale, which evaluates a child’s capacity to interact with peers, engage in play, and work autonomously; and the school scale, which assesses academic performance. Profile sheets were used for scoring, divided by sex, age, and type of scale, allowing the raw scores to be transformed into T-scores and comparison with normative data derived from reference samples of the same age. The retest reliability for the skills scale was 0.90, while the internal consistency was supported by an alpha coefficient ranging from 0.63 to 0.79 [19].

#### 2.4.5. Strengths and Weaknesses Questionnaire (SDQ-ITA, Version 4–10 Years for Parents and Teachers)

The Italian version of the “Strength and Difficulties Questionnaire—SDQ” [20] was used. The SDQ is a questionnaire composed of 25 items divided into five subscales: Hyperactivity, Conduct Disorders, Emotional Problems, Peer Relations, and Prosocial Behaviors. The same questionnaire can be filled out by both teachers and parents and allows the individual to obtain much information about the child’s behaviour. The ratings range from 0 (not true) to 1 (fairly true) to 2 (certainly true). For all scales, a higher score corresponds to a higher level of difficulty, with the exception of the Prosocial Behaviours scale, in which a high score indicates a significant presence of positive behaviours. Calibration of the SDQ-ITA was obtained from a sample of 528 elementary school students in the provinces of Vicenza and Venice. The SDQ demonstrated good psychometric properties, with a five-factor structure and good internal consistency for the five subscales (Cronbach’s alpha between 0.73 and 0.89). The SDQ percentile scores were reported to allow the use of the questionnaire both as an information sheet on the psychological difficulties of students and as a screening tool for schools (to identify cases of behavioural and emotional difficulties).

## 3. Results

### 3.1. The Starting Digital Age and the Type of Use of Digital Devices

From the frequency distribution of the responses on the age of first use of technology given by the parents, it emerged that the average age at which the children in our sample began to use technology was 5.91 years (SD = 2.25). The children used a digital device for the first time, mainly between the ages of six and seven (37.3%), 20.4% between the ages of eight and nine, and 28.1% between the ages of three and five. A minority of children started before the age of two (9.3%) and only 4.8% between the ages of nine and eleven years. The device most used by the children in the sample along with parental reports was the tablet (49.8%), followed by the smartphone (35.4%), and the personal computer (PC) (12.2%). E-book readers were used less frequently (2.6%). Children aged 7–11 years old (N = 110) self-reported 73.6% of tablet use, followed by smartphones (64.2%), PC (58.2), and e-book readers (9.8). The average time in minutes of daily use of the tablet reported by parents in their children was 22.86 (SD = 33.62), of PC 15.68 (SD = 26.38), of smartphone 8.87 (SD = 24.43), and of e-book 1.23 (SD = 7.11). The children reported a mean daily tablet use of 27.80 min (SD = 35.39), followed by PC (Mean = 19.32; SD = 23.12), smartphone use (Mean = 11.11; SD = 21.88), and e-book readers (Mean = 2; SD = 9.68). The parental and child reports on the mean time of usage of the different technological devices were very in agreement, with no significant paired t-test that identified mean differences (*p* > 0.05). The activities reported by parents as the most frequently performed by children with digital devices were recreational activities (M = 3.15; SD = 0.72, range: 1–5) such as listening to music (48.1%), watching TV, videos, or cartoons (42.4%) and games for fun only (for example, Angry Birds, Fruit Ninja, etc.) (39.2%). The second category of activities observed by parents on their children is related to creative activities (M = 2.14; DS = 0.90; range: 1–5), followed by educational activities (M = 1.80; DS = 0.77; range: 1–4), with only 28.7% using them. Children self-reported their most frequent recreational activities (M = 3.27; SD = 0.84; range: 1–5), followed by creative ones (M = 2.45; SD = 0.69; range: 1–4.50), and educational ones (M = 1.64; SD = 0.56; range: 1–3).

A series of paired *t*-tests identified a significant difference in the means of creative activities in the child-reports compared to the parent-reports (t_109_ = −8.86; *p* < 0.001), with children reporting a higher frequency (M = 2.45; SD = 0.69) than their parents (M = 1.89; SD = 0.76). Table 4 shows a brief summary of these results.

### 3.2. Impact of Sociodemographic Variables on the Time of Use of Technology and the Typology of Activities

A series of ANOVAS were performed on the time children spent using digital devices at their age (grouped by age group). The analysis of this sample showed a significant effect of the age of the children on the frequency of tablet use (F_307,310_ = 2.85; *p* = 0.038), smartphone use (F_301,304_ = 4.38; *p* = 0.005) and e-books (F_308,311_ = 6.05; *p* = 0.001), while there was no significant effect of the age of the children on the frequency of PC use (Table 5). In particular, following the application of the post hoc analysis with the Bonferroni method (Table 6), it was observed that children in this sample in the 8–9 age group used the tablet significantly more than those in the 3–5 age group (*p* = 0.023); children aged 10–11 years used the smartphone significantly more than both children in the 3–5 age group (*p* = 0.012) and those in the 6–7 age group (*p* = 0.046); and finally, that older children used e-books significantly more frequently than younger children (*p* = 0.009), but also than the other age groups examined.

The ANOVAS analyses showed a significant effect of the age of the children on recreational activities (F_310,313_ = 7.48; *p* = 0.0001) and Internet use (F_310,313_ = 9.99; *p* = 0.0001) in this sample. Following the application of the post hoc analysis with the Bonferroni method, it was observed that younger children performed recreational activities and used the Internet significantly less than older children (Table 7).

**Figure 1:**
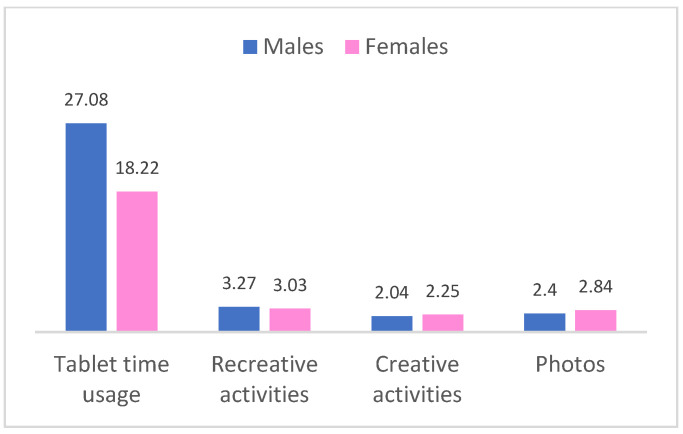
Comparison of the time spent and type of activity on digital devices between boys and girls.

A series of *t*-tests for independent samples were performed between the child gender variable and the variables relating to the time spent using technology and then between the child gender variable and the variables relating to the activities carried out with digital devices. The tablet use time was significantly different depending on the gender (t_309_ = −2.34; *p* = 0.02), and recreation (t_312_ = −2.95; *p* = 0.003), creative activities (t_312_ = 2.10; *p* = 0.0037), and photos (t_312_ = 3.84; *p* = 0.0001) were different depending on gender. Figure 1 shows the results for these participants.

### 3.3. Associations Between Parental SES and Time and Typology of Use of Technological Devices

The use of tablet time by children was significantly associated with the fathers’ school years (r = −0.21; *p* = 0.034) in this sample, indicating that a higher educational qualification of fathers was associated with a lower use of tablets by the child, and vice versa. The father’s school years were also significantly associated with the performance of recreational activities with digital devices (r = −0.32; *p* = 0.001), so a higher educational qualification of the father corresponded to a lower use of recreational activities with digital devices by the child. A significant effect of the family’s economic situation on the time spent using a smartphone emerged (F_302,305_ = 2.94 *p* = 0.054), while in the other typology of device no significative difference was obtained (Table 8). Following the application of the post hoc analysis with the Bonferroni method, it can be observed that parents with a low perceived economic condition declared that their children used the smartphone for more time than those with a medium economic condition (M = 18.38 SD = 49.27 against M = 7.62 SD = 15.58). Table 9 shows a brief summary of these results.

### 3.4. Behavioral Difficulties and Adaptive Skills Along Children’s Sociodemographic Factors

**Figure 2:**
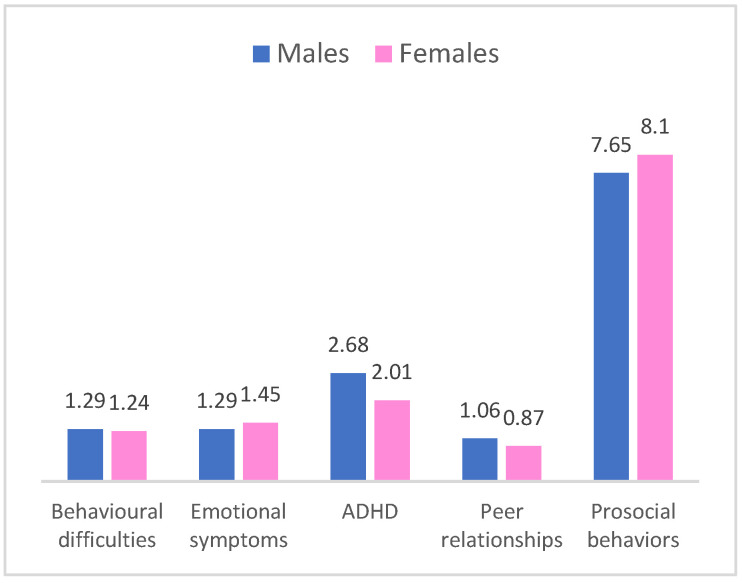
The relative frequencies of the T-scores of the global SDQ and scale scores along child’s gender.

A statistically significant difference was found for the ADHD scale by sex (t_312_ = −3.13; *p* = 0.002), such that boys had higher scores on the ADHD scale than girls (M = 2.68; SD = 2.08 versus M = 2.01; SD = 1.74). Therefore, we can state that in this sample, boys had more difficulties with attention and hyperactivity difficulties than girls. Furthermore, a statistically significant difference appeared between boys and girls on the Prosocial Behaviours Scale (t_312_ = 2.78; *p* = 0.010) as well, such that girls had higher average scores on the Prosocial Behaviours Scale than boys (M = 8.10; SD = 1.58 versus M = 7.65; SD = 1.53) (Figure 2).

An ANOVA analysis was performed to determine whether the child’s age had a significant effect on the SDQ scales in this sample. The Behavioural Problems Scale score differed significantly with age (F_310,313_ = 3.61; *p* = 0.014). After applying post hoc analysis with the Bonferroni method, it was observed that children aged 3–5 years had significantly higher scores on the Behavioural Problems Scale than those aged 10–11 years (mean difference = 0.72; *p* = 0.01); furthermore, it was observed that the children aged 6–7 years had higher scores on the Prosocial Behaviours Scale than those aged 3–5 years (mean difference = 1.10; *p* = 0.002). This result indicates that as age increases, behavioural problems decrease, and prosocial behaviours increase.

### 3.5. Associations Between Usage Time of Technical Devices and Psycho-Physical Health (Sleep and Behavioral Difficulties)

Parents declared an average of 9.55 (SD = 0.55) hours of sleep per night in their children. Pearson’s correlations were performed to evaluate whether the use time of technological devices was significantly associated with sleep duration. Analysis of the temporal patterns of digital device usage revealed that children predominantly engaged with technology in the late afternoon, specifically in the hours preceding dinner (69.1%), whereas a smaller proportion (10.8%) utilised these devices before bedtime. A significant correlation was found between computer usage time and sleep duration (r = −0.13, *p* = 0.019) in this sample, which indicated a decrease in sleep duration as the frequency of PC use increased, and vice versa.

**Figure 3:**
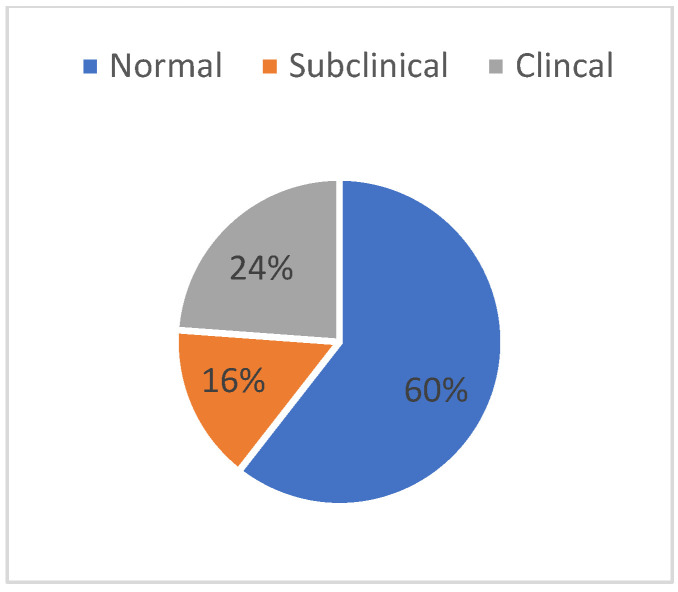
Relative frequencies of the cut-off in CBCL Total score referred to the children in the sample.

To evaluate children’s level of adaptation, parents were asked to complete the CBCL questionnaire in the section related to the skill scale. The sample size included 109 participants. In fact, this cohort of participants belonged to the initial data collection related to the project, and this questionnaire was no longer administered. Three different skill scores emerged from the scoring: one for the Activity scale, one for the Sociality scale, and one for the School scale. Subsequently, the overall skill score was calculated, and the cut-off levels adopting T-score conversion are shown in Figure 3.

Pearson’s correlations were performed between variables related to the time spent using technology and activities carried out with digital devices and the scores of the CBCL competency scales (Activity scale, Sociability scale, School scale, and Total score).

The Social Skills scale score significantly correlated with both the frequency of smartphone use (r = −0.024; *p* = 0.016) and the frequency of PC use (r = −0.33; *p* = 0.001). As the frequency of smartphone and PC use increased, the social skills scale score decreased and vice versa.

**Figure 4:**
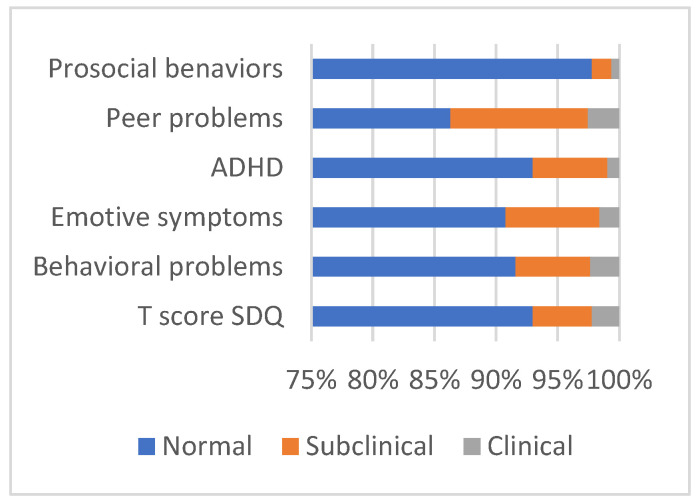
Relative frequencies of the cut-off T-scores of the SDQ global and scale scores referred to the children in the sample.

The analysis of the difficulty scores of the children adopting SDQ showed that 93% of them are normal, being below the 80th percentile; a total of 4.8% fall within the subclinical range, while 2.2% are considered clinical subjects as they are above the 90th percentile. Figure 4 shows the frequencies cutoff scores along the various SDQ scales.

The frequency of tablet use was significantly associated with the School scale score (r = −0.24; *p* = 0.012); therefore, a lower score on the School scale was associated with greater tablet use, and vice versa. Furthermore, it emerged that tablet frequency use was also significantly associated with the total CBCL score (r = −0.19; *p* = 0.048), so a higher total competency score corresponds to lower tablet use, frequency, and vice versa.

## 4. Discussion

In contemporary society, children are immersed in an environment where technology permeates every facet of their lives. According to the American Academy of Pediatrics (AAP) [21], children engage with digital devices more frequently than any other activity. Consequently, recommendations have been proposed regarding children’s use of technology, including limiting screen exposure to less than two hours per day [9,22]. This study aimed to deepen the issues related to the use of digital tools by children and the connection between the use of technology and adaptation, and the emotional and behavioural difficulties of children.

The first objective was to investigate the use of digital devices by children, analysing the average age at which they begin to use technologies; the digital devices they prefer to use between PC, smartphones, tablets, and e-books; the frequency of daily use for each technological tool; and the type of activities they carry out most when using technologies. The findings indicate that the average age at which children begin engaging in technology is approximately six years. This is earlier than that reported in previous studies, which identified seven years as the typical age for initial Internet use [23] and ten years for the first acquisition of a mobile phone [23,24]. The average age at first use has been reported to decrease, and should be cautiously established. Future analyses involving subsequent cohorts of children should aim to compare the current results with future data. In this study, the device most commonly used by children was the tablet, followed by the smartphone and the computer, while the e-book was still rarely used. Therefore, it seems that the use of smaller and more portable devices, such as tablets and smartphones, is currently preferred to the use of the computer, used for DVD and video games in recent years [25]. A recent study in 2023 by the Save the Children Association (32.6% between the ages of 6 and 10) confirmed that the percentage of daily use of small portable devices detected in our study is higher [1].

The activities performed most frequently by children in this study were recreational, including listening to music, playing games for fun, and watching videos, TV, and cartoons on digital devices, as reported in 2012 [25], even if among the activities that have increased are listening to music and using the camera to take photos or selfies, probably following the new trend of social networks and influencers. Parents and children aged more than 6 years old seemed not agree in their reports of favorite digital devices and in their mean time of use, while some different perceptions were found in creative activities more declared by children. This result should be taken with caution, because this study is explorative and it is necessary to better study this phenomenon.

The second objective was to understand whether the use of technological devices differed depending on the sex or age of the child. Regarding the age of children, the results of this study agree with the literature which shows an increase in the time spent using digital devices as age increases [23]. Children’s age was also found to be relevant for the performance of recreational activities and for the use of the Internet; specifically, older children (8–11 age group) perform recreational activities and use the Internet to a signifi-cantly greater extent than younger children (3–7 years). In terms of children’s gender, it was observed that, on average, boys exhibited a greater affinity for technology than girls.

However, a statistically significant difference was identified only in the duration of tablet usage, with boys spending more time in front of the tablet screen. Cannoni et al. [8] note that parents tend to encourage technological engagement more in their male children, and some fathers specifically impart an interest in technology to their sons. Future studies could examine whether and how the use of digital devices by both parents affects their children’s use of technology and whether any gender differences in parents’ use of digital tools affect their children’s use. Specifically, fathers’ higher educational qualifications and high economic conditions reduced tablet use time and recreational activities. The literature confirms this result, explaining that parents with higher education and good economic conditions exercise greater control over their children’s use of technology, as they are more aware of the associated risks [26,27,28].

The third objective was to identify possible associations between the use of technological devices and sleeping and behavioral difficulties. A decrease in sleep duration was recorded as the time spent using the computer during the day. These data are in line with the literature, which found a negative association with sleep hours, not only for the use of video games, smartphones, and television, but also for the use of computers [29]. The same result was also reported by Twenge et al. [22], who found a significant association between the time spent using digital devices, both portable and non-portable, and sleep duration in children under ten years of age.

The fourth objective was to assess the children’s overall level of adaptation, showing that they had a predominantly normal level, while approximately a quarter of the children were placed in the clinical range. With respect to technology use time, it has emerged that greater smartphone and computer use time is associated with poorer participation in extracurricular group activities, fewer friends, and worse behaviour toward significant others (parents, brothers/sisters, friends).

The findings align with two predominant theories that elucidate the relationship between technology use and social and relational dynamics. One theory posits that time spent engaging with technology detracts from time that could be allocated to social activities, thereby heightening the risk of social isolation or depression [30]. Conversely, other theories suggest that young individuals with actual or perceived social skill deficits are more inclined to utilise technology to compensate for their challenges [31,32]. Additionally, it is evident that children who frequently use tablets tend to exhibit lower academic performance and encounter greater academic difficulty. This observation is consistent with Peas [9], who reported that the use of entertainment media negatively affects children’s academic outcomes, primarily because such media are often used concurrently with homework.

With respect to total behavioral and emotional difficulties, most children classified in the clinical range appear to have problems with peers, emotional symptoms, and behavioral problems, while a small portion of children show symptoms of hyperactivity, inattention, and problems with prosocial behavior. As children grow, behavioural problems decrease and prosocial behaviors increase. In terms of gender differences among children, it is apparent that boys exhibit more ADHD symptoms than girls, while girls demonstrate a higher prevalence of prosocial behaviors than boys. Regarding technology usage, it has been observed that increased tablet use is associated with heightened behavioral, attentional, and hyperactivity challenges. This result is in line with the literature showing an association between externalizing symptoms, including inattention, hyperactivity, impulsivity, and high rates of technology use [30,33].

It is essential to consider not only the duration of technology use, but also the manner in which it is employed, the types of activities engaged in, and whether these activities are conducted individually or collaboratively with peers. In this context, parents have reported that technology offers benefits for their children’s intellectual, social, physical, and emotional development, thereby enhancing educational growth through educational activities, while simultaneously facilitating peer interaction [17]. It is important to emphasise that the manner of technology use is significant rather than merely the duration of use.

### 4.1. Strengths and Limits

This study involved a large and heterogeneous sample with respect to sociodemographic conditions; therefore, it can be considered partly representative of the population of interest. Nevertheless, the sample is exclusively derived from north-east Italy; thus, it would be valuable to examine the same phenomenon in other regions of the Italian Peninsula. The data collected were varied and extensive, enabling the correlation of numerous variables identified within the same sample, thereby facilitating various research possibilities. However, the study design was cross-sectional and restricted causal inferences. Although associations can be observed, it is not feasible to ascertain the directionality of the effects between technology use and behavioural difficulties. Furthermore, data collection was carried out over three years, allowing comparison between the different cohorts that participated in the survey and evaluation of how the same phenomenon changed over time. Since this is a rapidly evolving field, the literature available on this topic may already be outdated. It is important to note the huge variability in this issue due to the rapid changes in children’s digital habits, especially post-pandemic COVID-19. However, an important strength is that this study evaluates the agreement between the responses of parents and those of their children in order to have a multi-informant perspective.

### 4.2. Recommendations for Future Research

Future research could use multiple data collected to relate other variables of interest, expand the number by involving other areas of Italy, and compare these results with those obtained from the questionnaires filled out by teachers. This information and future studies could be informative for establishing preventive and educational programs for children, parents, and teachers. The directions could be attention to the time facing the screen, amelioration of parental control, and the ability of children to regulate their use, not excluding other important social activities and improving the educational activities adopting technical devices.

## 5. Conclusions

Children began to use technological devices when they were about 6 years old, and some earlier in their life cycle. This is earlier than that reported in previous studies, which identified seven years as the typical age for initial Internet use [23] and ten years for the first acquisition of a mobile phone [23,24]. The tablet was the most used device according to children’s and parent’s reports, followed by smartphone and PC, and the most frequent activities were recreational. It seems that the use of smaller and more portable devices, such as tablets and smartphones, is currently preferred to the use of the computer, used for see videos or playing video games in recent years [25], an increasing trend compared to the Save the Children Association’s recent study [21].

Parents and children agreed on the time spent with the technological devices reports, while they discorded on the means of creative activities use, with children reporting a higher frequency than their parents. The studies on comparing parent and child reports on their quantity and quality of use are missing and these results could be confirmed by other research. Boys used tablets for a longer mean time than girls and had more ADHD problems. Cannoni et al. [8] note that parents tend to encourage technological engagement more in their male children, and some fathers specifically impart an interest in technology to their sons. It is important to take into consideration the educative role of parents and especially fathers, mainly in the male children use to prevent also their possible ADHD difficulties. Children aged 8–9 years used the tablet significantly more than those in the 3–5 age group and children aged 10–11 years used the smartphone significantly more than the other ages groups. Targeted and preventive interventions should be directed towards the children aged 8–9 for the tablet quality of use and towards children aged 10–11 years for the smartphone.

The frequency of tablet use was associated with children’s behaviorual problems, especially ADHD. PC usage time was negatively associated with sleep hours duration, in line with the literature [22,29], while smartphone time of use was more frequent in families who reported low economic conditions. Fathers with higher levels of education seemed to be associated with children that used the tablet less, as confirmed by other studies on parental socio-economic influences [26,27,28].

## Figures and Tables

**Table 1:** Sociodemographic information of children.

		Frequencies	%
Gender	Females	150	47.8
	Males	164	52.2
	Total	314	100
Age	3–5 years	76	24.2
	6–7 years	90	28.7
	8–9 years	71	22.6
	10–11 years	77	24.5
	Total	314	100
School	pre-schooler	48	15.3
	1st primary school	23	7.3
	2nd primary school	31	9.9
	3rd primary school	62	19.7
	4th primary school	72	22.9
	5th primary school	78	24.8
	Total	314	100
		Mean	SD
Child’s age		7.86	2.02

**Table 2:** Sociodemographic information of parents.

	Respondent Parent				Partner
	Frequencies		%	Frequencies	%
Role	Mothers	253	80.6		
	Fathers	61	19.4		
	Total	314	100		
Level of education	Primary school	0	0	0	0
	Secondary school 1st grade	27	8.6	59	18.8
	Secondary school 2nd grade	159	50.6	158	50.3
	Degree 1st level	24	7.6	10	3.2
	Degree 2nd level	70	22.3	60	19.1
	Post-degree	34	10.8	23	7.3
	Missing	0	0	4	1.3
	Total	314	100	310	100
Current job	Part-time	129	41.1	70	22.3
	Full-time	134	42.7	221	70.4
	Looking for a job	40	12.7	18	5.7
	Missing	11	3.5	5	1.6
	Total	314	100	314	100
Job hours/week	50 or more	24	7.6	53	16.9
	40–49	76	24.2	165	52.5
	30–39	79	25.2	57	18.2
	20–29	81	25.8	16	5.1
	10–19	29	9.2	8	2.5
	0–9	13	4.1	10	3.2
	Missing	12	3.8	5	1.6
	Total	314	100	314	100
N. siblings	0	75	23.9		
	1	108	34.4		
	2	103	32.8		
	3	15	4.8		
	4	12	3.8		
	5	1	0.3		
	Missing	0	0		
	Total	314	100		
Perception of economic condition	Low	34	10.8		
	Average	191	60.8		
	High	89	28.3		
	Total	314	100		
	Mean		SD		
Parental Age	42.74		5.36		

**Table 3:** Parent-report questionnaire on digital experience for children aged 3–11.

Section	Items	Istructions	Select Responses
Order	1	Rank the following technological devices according to your child’s use of them (1 = most used; 4 = least used)	Tablet, Smartphone, PC, E-book reader
Frequency	2 3 4 5	How often does your child use the tablet? How often does your child use the PC? How often does your child use the smartphone? How often does your child use the e-book reader?	Never 1–15 min per day 16–30 min per day 31–60 min per day 1–2 h per day 2–3 h per day 3–4 h per day Over 4 h per day
Digital experience	6	How long has your child been using these technological tools? How old was your child when they started?	Very recently Quite a while ago A long time ago A very long time ago
Type of activity	7	What kind of activities does your child do when using them? (a) Memory game or matching game (b) Math/calculation games (c) Interactive reading books (d) Writing games (e) History/geography games (f) Exclusively fun games (e.g., Angry Birds, Fruit Ninja, etc.) (g) I use apps/programs for drawing/coloring/painting (h) I use apps/programs for making music (not to listen to music, but to produce it) (i) I watch TV/cartoons/films (l) I listen to music (m) I use the Internet (n) I use the camera for photos or selfies (I use my tablet or smartphone to take photos)	Never Almost never Sometimes Frequently Very frequently

**Table 4:** Brief summary of type of use of digital devices comparing children’s and parent’s reports.

	Children	Parents	Agreement
Device most used (ordered)	Tablet, smartphone, PC, e-book reader	Tablet, smartphone, PC, e-book reader	YES
Devices’ mean time of usage	Tablet (22.86), PC (15.68) smartphone (8.87), e-book reader (1.23)	Tablet (27.80), PC (19.32) smartphone (11.11), e-book reader (2)	YES
Type of activities	Recreational (3.15), creative (2.14), educational (1.80)	Recreational (3.27), creative (2.45), educational (1.64)	NO for creative activities, YES for recreational and educational activities

**Table 5:** One-way ANOVA with DV, the time spent on digital devices and IV, the age of the children.

	F	df1	df2	*p*
Tablet	2.85	3	307	0.038
Smartphone	4.38	3	301	0.005
PC	1.48	3	307	0.220
E-book reader	6.05	3	308	0.001

**Table 6:** Bonferroni post hoc test with DV, the time spent on digital devices and IV, the age groups of children.

	Age Range A	Age Range B	Usage Mean A		Usage Mean B		Mean Differences (A-B)	*p* Value
			M	SD	M	SD		
**Tablet**	8–9	3–5	27.52	37.87	10.89	18.06	16.63 *	0.023
**Smartphone**	10–11	3–5	15.01	29.67	0.93	3.62	14.08 *	0.012
	10–11	6–7	15.01	29.67	3.78	13.17	11.23 *	0.046
**E-book**	10–11	3–5	4.14	13.47	0.001	0.001	4.14 *	0.009
	10–11	6–7	4.14	13.47	0.12	0.90	4.03 *	0.005
	10–11	8–9	4.14	13.47	0.45	2.85	3.69 *	0.002

* *p* < 0.05.

**Table 7:** Bonferroni post hoc test with DV, the type of activities on digital devices and IV, the age groups of children.

	Age Range A	Age Range B	Usage Mean A		Usage Mean B		Mean Differences (A-B)	*p* Value
			M	SD	M	SD		
**Recreational activities**	8–9	3–5	3.22	0.72	2.84	0.66	0.38 *	0.011
	10–11	3–5	3.38	0.71	2.84	0.66	0.54 *	0.000
	10–11	6–7	3.38	0.71	2.96	0.70	0.41 *	0.004
**Internet**	8–9	3–5	2.54	1.14	1.76	1.08	0.78 *	0.001
	10–11	3–5	2.89	1.08	1.76	1.08	1.10 *	0.000
	10–11	6–7	2.89	1.08	2.20	1.29	0.66 *	0.006

* *p* < 0.05.

**Table 8:** One-way ANOVA with DV, the time spent on digital devices and IV, the family’s economic condition.

	F	df1	df2	*p*
Tablet	2.21	2	307	0.11
Smartphone	2.94	2	301	0.05
PC	2.36	2	307	0.09
E-book reader	0.69	2	308	0.50

**Table 9:** Brief summary of type of use of digital devices along parental SES.

Children	Father
More time of use of tablet	Less schooling years
More time of use of smartphone	Low economic condition

## Data Availability

The research data have been submitted as supplementary material.

## Supplementary Materials

The following supporting information can be downloaded at: https://www.mdpi.com/article/10.3390/pediatric17050099/s1. The research data file has been submitted here.
