# Supplementary material for: Use of Technological Devices in Children Aged 3–11 Years: Possible Effects on Sleep and Behavioral Difficulties
Source: Pediatr Rep. 2025 Sep 29;17(5):99. doi: 10.3390/pediatric17050099 (PMC12566866; doi:10.3390/pediatric17050099)
Supplement: Supplementary file 1 [file pediatrrep-17-00099-s001.zip › pediatrrep-3639788-supplementary- Self-report child technology experience.pdf]

SELF-REPORT QUESTIONNAIRE ON DIGITAL EXPERIENCE FOR CHILDREN AGED 8-11

M. Tremolada and S. Bonichini, 2017 - DPSS – UniPd

We would like to understand how much and how you use technology.
Please answer the following questions honestly.

Type of technology

1. Which of these technological devices do you use the most?
   - ❑ Tablet
   - ❑ Cell phone/Smartphone
   - ❑ Computer (PC or Mac)
   - ❑ E-book reader (Kindle, Kobo, Tolino, etc.)

Frequency of use

2. How often do you use your tablet?
   - ❑ Never
   - ❑ 1–15 minutes per day
   - ❑ 16–30 minutes per day
   - ❑ 31–60 minutes per day
   - ❑ 1–2 hours per day
   - ❑ 2–3 hours per day
   - ❑ 3–4 hours per day
   - ❑ Over 4 hours per day

3. How often do you use the computer?
   - ❑ Never
   - ❑ 1–15 minutes per day
   - ❑ 16–30 minutes per day
   - ❑ 31–60 minutes per day
   - ❑ 1–2 hours per day
   - ❑ 2–3 hours per day
   - ❑ 3–4 hours per day
   - ❑ Over 4 hours per day

4. How often do you use your cell phone/smartphone?
   - ❑ Never
   - ❑ 1–15 minutes per day
   - ❑ 16–30 minutes per day
   - ❑ 31–60 minutes per day
   - ❑ 1–2 hours per day
   - ❑ 2–3 hours per day
   - ❑ 3–4 hours per day
   - ❑ Over 4 hours per day

5. How often do you use an e-book reader?
   - ❑ Never
   - ❑ 1–15 minutes per day
   - ❑ 16–30 minutes per day

- ❑ 31–60 minutes per day
- ❑ 1–2 hours per day
- ❑ 2–3 hours per day
- ❑ 3–4 hours per day
- ❑ Over 4 hours per day

Digital Experience

6. How long have you been using these technological tools?
- ❑ Very recently
- ❑ Quite a while ago
- ❑ A long time ago
- ❑ A very long time ago
- ❑ How old were you when you started?

Type of activity

7. What do you do when you use them?

(a) Memory game or matching game
- ❑ Never
- ❑ Almost never
- ❑ Sometimes
- ❑ Frequently
- ❑ Very frequently

(b) Math/calculation games
- ❑ Never
- ❑ Almost never
- ❑ Sometimes
- ❑ Frequently
- ❑ Very frequently

(c) Interactive reading books
- ❑ Never
- ❑ Almost never
- ❑ Sometimes
- ❑ Frequently
- ❑ Very frequently

(d) Writing games
- ❑ Never
- ❑ Almost never
- ❑ Sometimes
- ❑ Frequently
- ❑ Very frequently

(e) History/geography games
- ❑ Never
- ❑ Almost never
- ❑ Sometimes
- ❑ Frequently
- ❑ Very frequently

(f) Exclusively fun games (e.g., Angry Birds, Fruit Ninja, etc.)
- ❑ Never
- ❑ Almost never
- ❑ Sometimes
- ❑ Frequently
- ❑ Very frequently

(g) I use apps/programs for drawing/coloring/painting
- ❑ Never
- ❑ Almost never
- ❑ Sometimes
- ❑ Frequently
- ❑ Very frequently

(h) I use apps/programs for making music (not to listen to music, but to produce it)
- ❑ Never
- ❑ Almost never
- ❑ Sometimes
- ❑ Frequently
- ❑ Very frequently

(i) I watch TV/cartoons/films
- ❑ Never
- ❑ Almost never
- ❑ Sometimes
- ❑ Frequently
- ❑ Very frequently

(l) I listen to music
- ❑ Never
- ❑ Almost never
- ❑ Sometimes
- ❑ Frequently
- ❑ Very frequently

(m) I use the Internet
- ❑ Never
- ❑ Almost never
- ❑ Sometimes
- ❑ Frequently
- ❑ Very frequently

(n) I use the camera for photos or selfies (I use my tablet or smartphone to take photos)
- ❑ Never
- ❑ Almost never
- ❑ Sometimes
- ❑ Frequently
- ❑ Very frequently
